# Research on the construction of an AI diagnostic model for plus disease of retinopathy of prematurity based on cross-center fusion datasets

**DOI:** 10.3389/fped.2026.1765353

**Published:** 2026-03-24

**Authors:** Xiqianru Zhang, Huichun Liang, Ruifeng Wang, Rouqing Wu, Xiao Shen, Yuemei Zhang

**Affiliations:** 1 The First School of Clinical Medicine, Lanzhou University, Lanzhou, Gansu, China; 2 School of Medical Informatics and Engineering, Gansu University of Chinese Medicine, Lanzhou, Gansu, China; 3 Key Laboratory of Dunhuang Medical and Transformation, Ministry of Education of the People’s Republic of China, Gansu University of Chinese Medicine, Lanzhou, Gansu, China; 4 Department of Ophthalmology, The First Hospital of Lanzhou University, Lanzhou, Gansu, China

**Keywords:** artificial intelligence (AI), convolutional neural network (CNN), cross-center datasets, retinopathy of prematurity (ROP), ROP plus disease

## Abstract

**Purpose:**

Retinopathy of prematurity (ROP) is a leading cause of blindness in infants. Early and accurate screening is essential. Current deep learning systems can help, yet their accuracy drops when used on different population. We aimed to find the best deep learning model for plus disease in ROP and to test it on a multi-center dataset.

**Methods:**

We built a cross-center retinal image database by merging public and private sets (FARFUM-RoP, HVDROPDB, LAN-RoP, Preterm infants <34 weeks GA from three tertiary NICUs, 2635 images). Nine types models were compared: ResNet34, ResNet50, DenseNet, Inception, MobileNet, VGG16, VGG19, EfficientNet, and Swin-Transformer. We compared FLOPs, parameter count, accuracy, recall, precision, and F1 score. ResNet50 showed the best balance and was kept. Ten-fold cross-validation was run on FARFUM-RoP alone, LAN-RoP alone, and their combined set.

**Results:**

Across the three diagnostic tasks, the ResNet50 algorithm attained area-under-the-curve (AUC) values of 0.97, 0.95 and 1.00 (95% CI 0.94–0.99, 0.91–0.98, 0.97–1.00) for Normal, Pre-plus and Plus disease, respectively. When trained on the consolidated multi-centre cohort, the model achieved optimal overall performance, delivering an accuracy of 92.60%, recall of 92.58%, precision of 92.69% and F1-score of 92.60%—all metrics surpassing those obtained with any single-centre training set.

**Conclusion:**

Compared with single-centre training, the cross-centre fusion strategy significantly enhanced the generalisability of the artificial-intelligence model, yielded superior diagnostic indices, and improved diagnostic accuracy for infants from diverse demographic backgrounds.

## Introduction

Retinopathy of prematurity (ROP) mainly affects preterm and low birth weight infants and is a major cause of childhood blindness (1). As more premature babies survive, the incidence of ROP is rising (2), placing a heavy burden on families and society. Timely screening and prompt treatment can sharply reduce the risk of severe ROP and blindness (3). Yet there are too few ophthalmologists trained to read ROP images, especially in resource-limited regions (4). A fast, scalable tool for diagnosis is urgently needed. Recent advances in artificial intelligence, particularly convolutional neural networks (CNN) for medical imaging, have opened new avenues for automated ROP diagnosis (5–7). Most current models, however, are trained and tested on single, homogeneous populations (8–10). Retinal structure and lesion features differ among ethnic groups (11), so these models lose accuracy when applied to new racial or geographic settings. This performance degradation reflects limited model generalizability across populations and compromises diagnostic reliability (12–16). Fusing data from multiple centers constitutes an effective strategy to enhance such generalizability (17–20). To address this issue, we aimed to improve the generalizability of ROP diagnosis across center. We systematically compared nine representative deep learning architectures, from classic VGGNet and ResNet to recent EfficientNet and Swin-Transformer. We measured FLOPs, parameter count, accuracy, recall, precision, and F1 score to quantify each model's performance. We then used two center distinct datasets, FARFUM-RoP and LAN-RoP, along with their merged set, and applied ten-fold cross-validation to study how data heterogeneity affects generalization. Our goal was to build an AI model for plus disease in ROP that leverages a cross-center dataset to enhance reliability. This work supports fair and robust ROP screening and offers new directions for AI in ophthalmology.

## Methods

**Figure 1:**
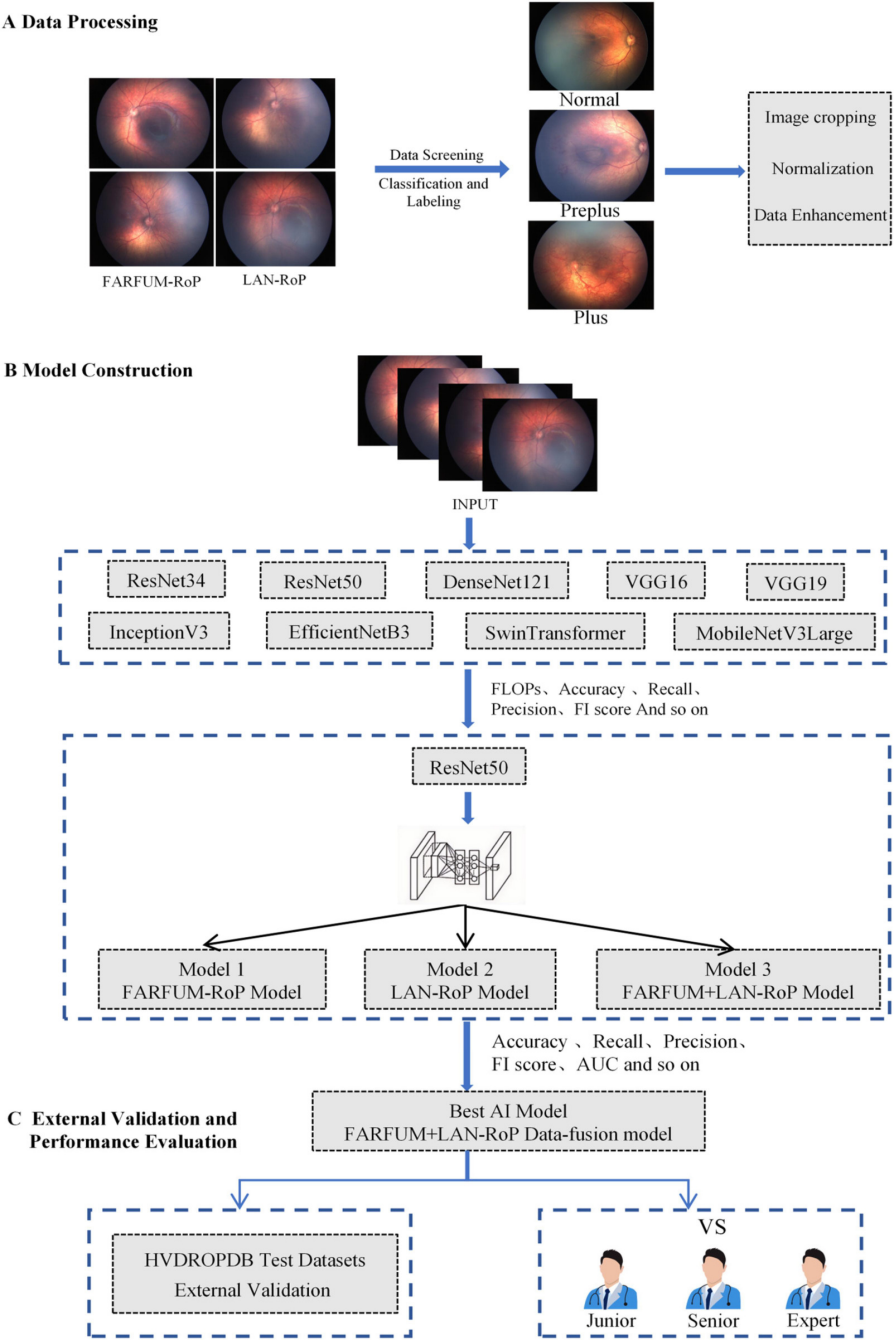
Research flowchart. **(A)** Images from FARFUM-RoP and LAN-RoP were cropped, screened, normalized, augmented, and labeled; **(B)** ResNet50 was selected among nine backbones and trained on single and fusion dataset; **(C)** externally validated on HVDROPDB and assessed by junior, senior, and expert ophthalmologists.

This study had three parts: dataset preparation, model building and tuning, and external validation. Figure 1 shows the full workflow.

### Dataset preparation

We used two public datasets, FARFUM-RoP and HVDROPDB, and one private datasets, LAN-RoP.

**FARFUM-RoP:** This public datases from Farabi and Ferdowsi Universities in Mashhad, Iran, and covers Caucasian infants (21). It includes 1,533 images from 68 preterm infants (birth weight <2,000 g, gestational age <34 weeks). RetCam captured 2–12 images per infant in five fields: posterior pole, superior, inferior, nasal, and temporal. Mydriasis was achieved with three drops of 0.5% phenylephrine and 0.5% tropicamide given every ten minutes. Five senior pediatric ophthalmologists labeled each image as normal, pre-plus, or plus. Although the original paper did not report inter-rater reliability coefficients, the expert-consensus labels provided by the dataset have been widely adopted as reference standards in multiple ROP-AI studies; therefore, we followed its annotation protocol without additional reassessment.

**LAN-RoP:** This private retrospective set covers infants screened at the The First Hospital of Lanzhou University from January 2019 to March 2025. It includes 1,002 images from 52 preterm infants (birth weight <2,000 g, gestational age <34 weeks) were included. No infant had received bevacizumab or laser before imaging. Mydriasis used compound tropicamide. RetCam wide-field systems obtained 2–15 images per eye in the same five fields. Three ophthalmologists (junior, attending, and senior) labeled every image as normal, pre-plus, or plus following the third edition of the International Classification of Retinopathy of Prematurity (22). They had joint training and removed poor-quality images. We used the mean across-category accuracy of the three ophthalmologists as a qualitative indicator of inter-rater agreement. The hospital Ethics Committee approved the study.

**Mixed dataset:** We merged FARFUM-RoP and LAN-RoP to create a larger, more diverse training set.

**HVDROPDB:** This public multimodal dataset was collected at H. V. Desai Eye Hospital in India from 2020 to 2023 (23). It contains 3,000 RetCam and Neo images. We used a subset (100 images) for external validation.

All images were preprocessed in the same way: resized, denoised, and contrast-enhanced to reduce equipment differences.

### Model selection and comparison

To find the best model, we ran all experiments on identical hardware and software: GPU:RTX 3,090(24GB) * 1, CPU:14 vCPU Intel(R) Xeon(R) Gold 6,330 CPU @ 2.00 GHz, PyTorch 2.3.0, Python 3.12. We trained on FARFUM-RoP with fixed hyperparameters (epochs, batch size, AdamW optimizer, and initial learning rate).Models included classic CNNs (VGGNet, ResNet, DenseNet, Inception, MobileNet), the efficient network EfficientNet, and the vision transformer Swin-Transformer (24–28). Each model started from ImageNet pretrained weights. We measured parameters, FLOPs, accuracy, recall, precision, F1 score, ROC curve, and AUC (29, 30). These metrics were derived from true positives (TP), true negatives (TN), false positives (FP), and false negatives (FN). The performance metrics were calculated as follows (Equations 1–4):

$$\mathrm{Accuracy}\;=\frac{\mathrm{TP}\;+\;\mathrm{TN}}{\mathrm{TP}+\mathrm{FP}+\mathrm{TN}+\mathrm{FN}}$$ (1)

$$\mathrm{Precision}\;=\frac{\mathrm{TP}}{\mathrm{TP}+\mathrm{FP}}$$ (2)

$$\mathrm{Recall}\;=\frac{\mathrm{TP}}{\mathrm{TP}+\mathrm{FN}}$$ (3)

$$F1\;=\;2\;*\;\frac{\mathrm{Precision}\;*\;\mathrm{Recall}}{\mathrm{Precision}+\mathrm{Recall}}$$ (4)

ROC curve plots the true positive rate (TPR) against the false positive rate (FPR) at every threshold. The area under this curve (AUC) gives a single number that shows overall model strength. An AUC close to 1 means strong classification. Parameters count tells us how large the model is and how much memory it needs. FLOPs count the floating point operations used in one forward pass. It is a hardware-free measure of model speed.

### Ten-fold cross-validation

Based on the comparison, we chose ResNet50 as the final backbone. Its skip connections ease gradient vanishing and let the network go deeper without adding too many parameters. ResNet50 has shown strong results in many medical image tasks. We ran three separate ten-fold cross-validation experiments to test the model. In each experiment we split the dataset into ten equal parts. Nine parts trained the model and one part served as validation. We rotated the validation part ten times so every image was used for both training and testing once. We report the mean of the ten runs. This approach reduces variance and gives a reliable estimate of model performance (Table 1).

**Table 1:** Ten-fold cross-validation experimental design.

Model	Ten-fold cross-validation	Test datasets
FARFUM-RoP Model	FARFUM-RoP	FARFUM-RoP Test + LAN-RoP Test
LAN-RoP Model	LAN-RoP	LAN-RoP Test + FARFUM-RoP Test
FARFUM + LAN RoP Model	FARFUM-RoP + LAN-RoP	LAN-RoP Test + FARFUM-RoP Test

Ten-fold cross-validation was performed on FARFUM-RoP, LAN-RoP, and their fusion dataset, with model performance evaluated independently on the FARFUM-RoP test set and the LAN-RoP test set.

### Statistical analysis

We used SPSS 29.0. Results are shown as mean ± standard deviation. We compared multiple groups with one-way ANOVA. For pairwise comparisons we used the LSD test. A *P* value below 0.05 was considered significant.

This study conforms to the TRIPOD-AI 2024 statement for reporting machine-learning-based diagnostic models (31).

## Results

### Model interpretability

**Figure 2:**
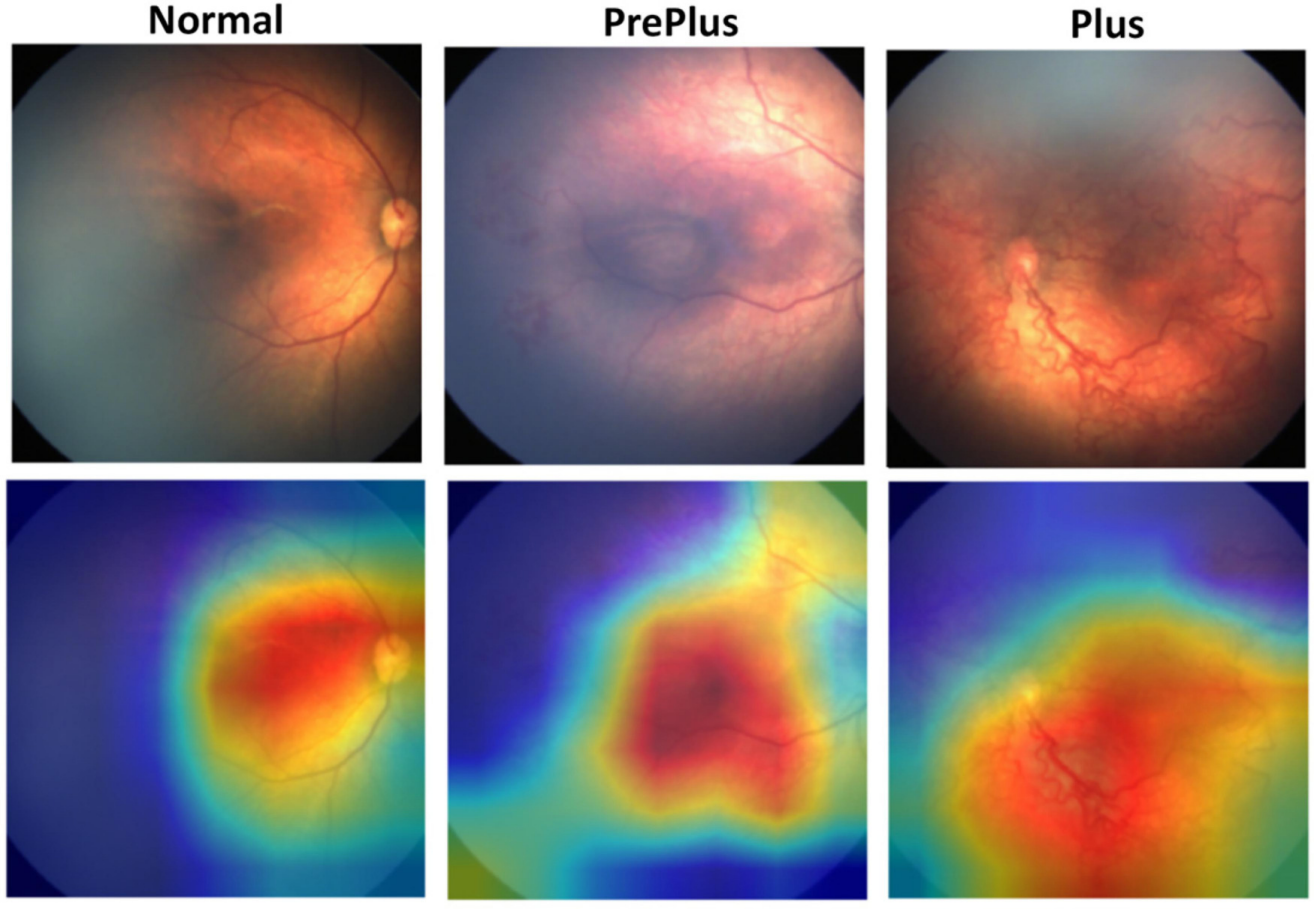
Heat map of the fundus image. The model highlights regions of interest in purple-to-red; deeper red indicates higher activation. The network concentrates on dilated, tortuous vessels in the posterior pole, aligning with clinical signs and confirming interpretability.

To clarify how the model makes decisions, we created heat maps (Figure 2). Each map marks the image regions that drew the model's attention. Warmer colors show higher activation, moving from purple to red. The maps reveal that the network focuses on the same signs clinicians use: dilated and tortuous vessels in the posterior pole. The heat-maps partially overlap with the posterior pole vessel regions, providing some qualitative support for the model's clinical relevance, though quantitative validation remains for future work.

### Model selection and comparison

**Figure 3:**
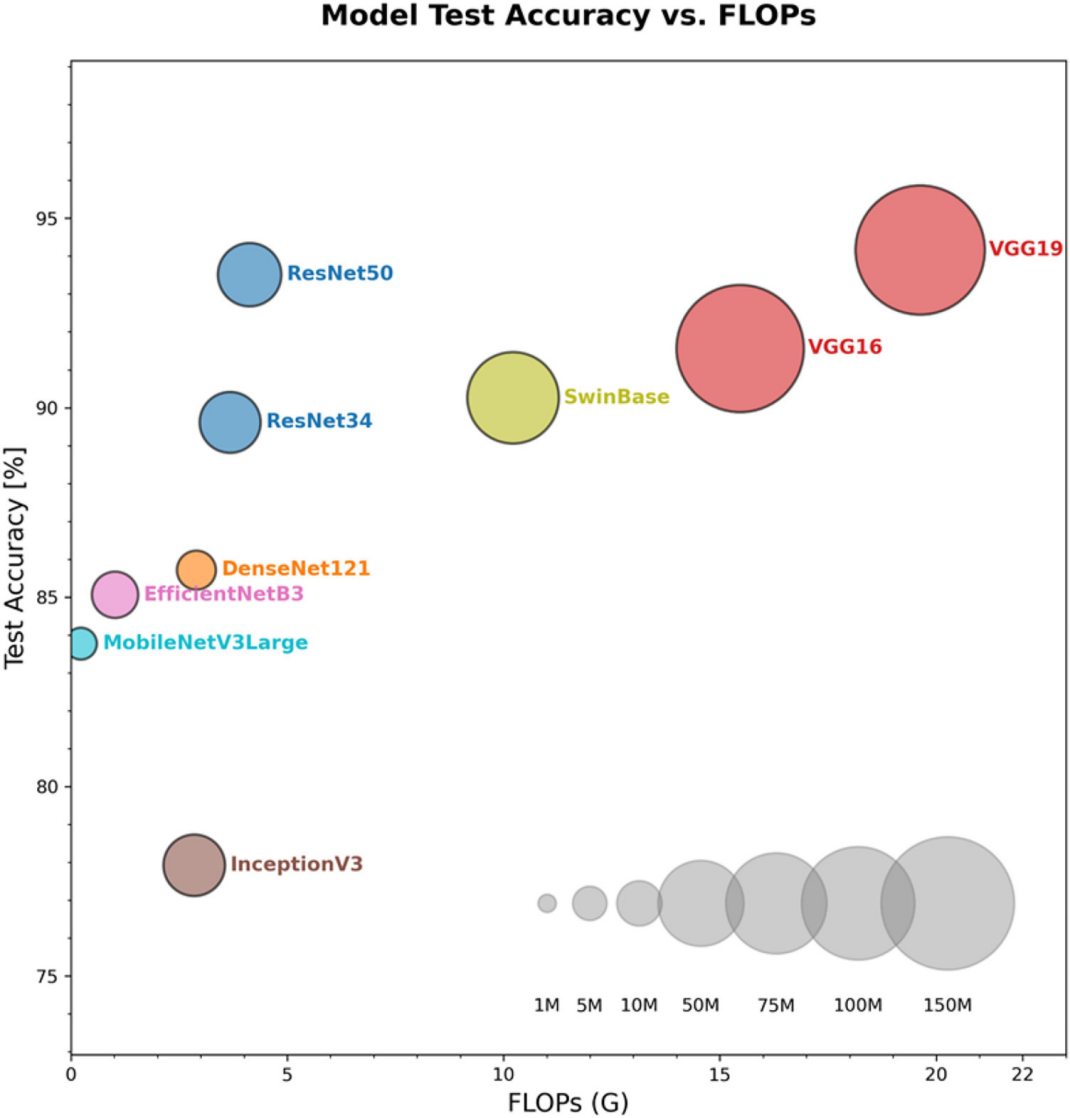
Comparison of FLOPs and accuracy rate of different deep learning models. The *x*-axis shows FLOPs (G), the *y*-axis shows test accuracy (%). ResNet50 achieves the highest accuracy at ≈4 G FLOPs, outperforming VGG variants and other lightweight networks while maintaining efficiency.

Figure 3; Table 2 show the test set results. VGGNet reaches reasonable accuracy but uses far more parameters and FLOPs. MobileNetV3Large and EfficientNetB3 are light yet give slightly lower accuracy. ResNet50 offers the best balance. It keeps high accuracy while keeping both FLOPs and parameter count moderate. We therefore chose ResNet50 as the backbone for the next steps.

**Table 2:** Comparison of parameters of different deep learning models.

Model	Accuracy (%)	Precision (%)	Recall (%)	F1-Score (%)	Total Parameters	FLOPs
ResNet34	89.61	89.58	89.61	89.59	21,286,211.00	3.68 GFLOPs
ResNet50	93.51	93.49	93.51	93.41	23,514,179.00	4.13 GFLOPs
DenseNet121	85.71	85.98	85.71	85.83	6,956,931.00	2.90 GFLOPs
VGG16	91.56	91.71	91.56	91.61	134,272,835.00	15.47 GFLOPs
VGG19	94.16	94.23	94.16	94.18	139,582,531.00	19.63 GFLOPs
InceptionV3	77.92	78.58	77.92	78.15	21,791,715.00	2.85 GFLOPs
EfficientNetB3	85.06	85.15	85.06	84.35	10,700,843.00	1.02 GFLOPs
SwinTransformer	90.26	90.32	90.26	90.29	58,716,291.00	10.22 GFLOPs
MobileNetV3Large	83.77	84.17	83.77	83.93	4,205,875.00	0.23 GFLOPs

VGG19 achieved the highest accuracy, precision, recall and F1 score, but required the most parameters and FLOPs. ResNet50 delivered 93.51% accuracy with only 4.13 GFLOPs, offering the best trade-off and was selected as the optimal model.

### Ten-fold cross-validation results

**Figure 4:**
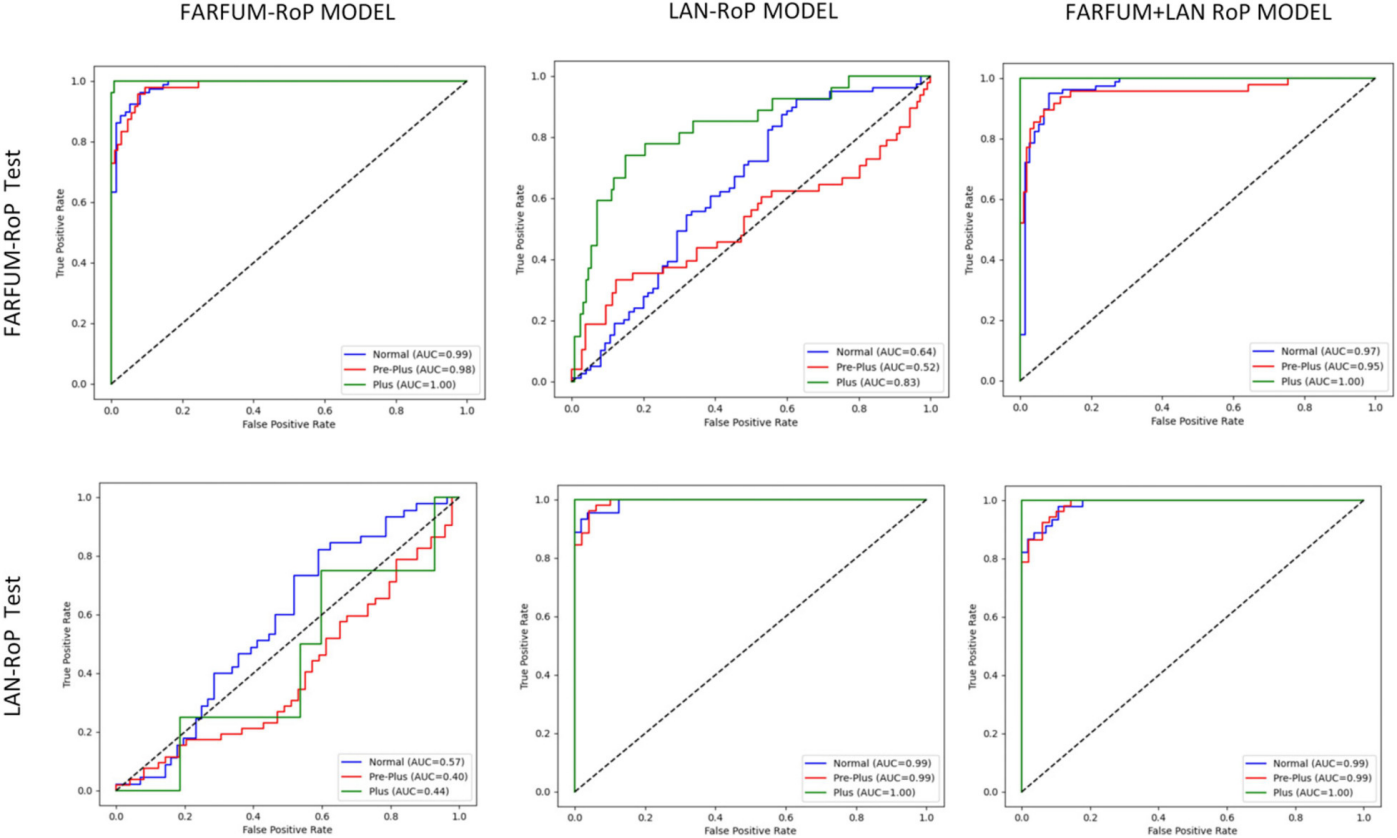
Comparison of ROC curves of three AI models on different test sets. Three models were trained via 10-fold cross-validation on different datasets and evaluated on the FARFUM-RoP and LAN-RoP test sets. The FARFUM + LAN-RoP fusion model produced superior ROC curves and higher AUCs across all test sets, whereas single-dataset models showed marked performance drops on cross-dataset testing, confirming that fusion training mitigates racial bias and enhances generalization.

We kept ResNet50 as the backbone and trained three models: FARFUM-RoP Model, LAN-RoP Model, and FARFUM + LAN-RoP Model. Each model went through ten-fold cross-validation on its own or the cross-dataset test set. Figure 4; Table 3 give the mean scores. The FARFUM + LAN-RoP Model reached the highest accuracy, recall, and precision and showed the smallest spread across folds. The single-set models scored lower and varied more, suggesting they learnt dataset-specific patterns and lost generalizability. The ROC curve of the merged model ran closest to the upper left corner and its AUC was near 1.

**Table 3:** Shows the average results of ten-fold cross-validation on different test sets.

Model	FARFUM-RoP Test				LAN-RoP Test			
	Accuracy（%）	F1 score（%）	Precision (%)	Recall (%)	Accuracy（%）	F1 score（%）	Precision (%)	Recall (%)
FARFUM-RoP Model	92.08 ± 1.50	92.04 ± 1.53	92.14 ± 1.52	92.08 ± 1.50	36.63 ± 2.80	35.05 ± 3.29	46.73 ± 4.80	36.63 ± 2.80
LAN-RoP Model	42.08 ± 4.51	40.14 ± 5.85	49.69 ± 5.01	42.08 ± 4.51	93.66 ± 1.89	93.65 ± 1.86	93.81 ± 1.85	93.66 ± 1.89
FARFUM + LAN RoP Model	92.60 ± 1.65	92.58 ± 1.64	92.69 ± 1.62	92.60 ± 1.65	94.55 ± 2.39	94.55 ± 2.39	94.59 ± 2.39	94.55 ± 2.39

Single-dataset models excelled on their own test set but dropped sharply when tested across datasets; the FARFUM + LAN-RoP fusion model sustained high accuracy, F1-score, and stability on both test sets, confirming that fusion training mitigates ethnic bias and enhances generalizability.

### AI vs. ophthalmologists

**Figure 5:**
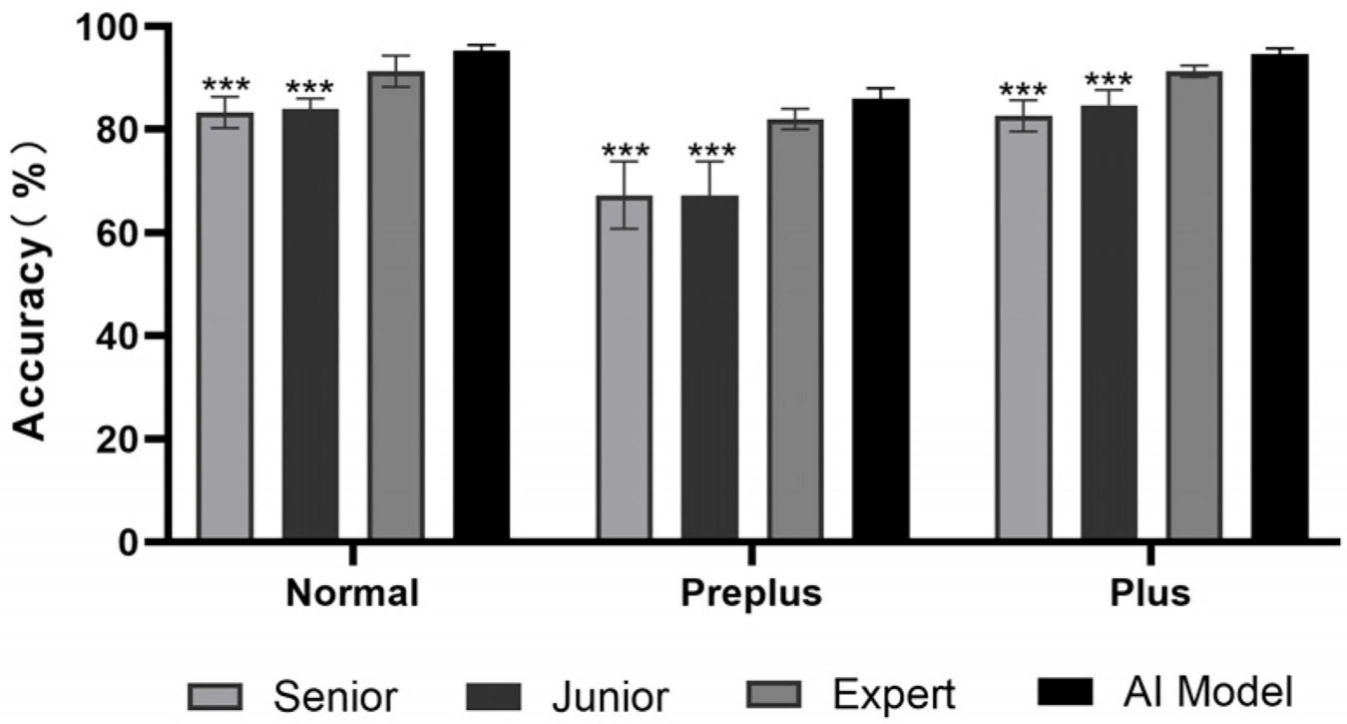
Comparison between AI models and ophthalmologists. The AI achieved 95%, 86%, 95% for Normal, Pre-plus, Plus, outperforming the average clinician 86%, 72%, 86%, and approaching expert performance. ***P < 0.001.

We compared the best model (FARFUM + LAN RoP Model) with three experienced ophthalmologists on the same test set. Average accuracies for the doctors were 86% for Normal, 72% for Preplus, and 86% for Plus. The AI model reached 95%, 86%, and 95%. Thus, AI model's accuracy surpassed the doctors, especially junior ones, and approached expert level. The model also processed images faster, making large-scale ROP screening feasible (Figure 5).

### External validation

**Figure 6:**
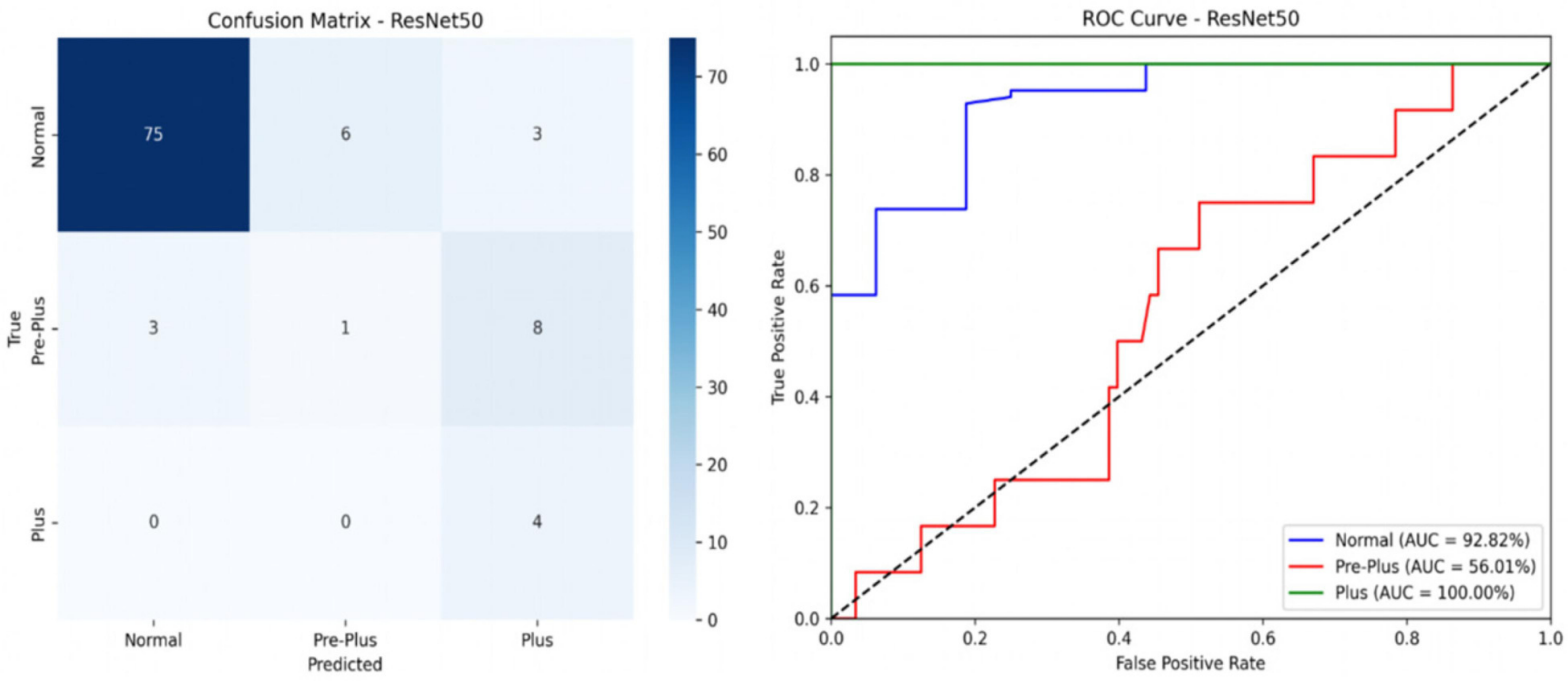
External validation results (matrix and ROC curve). The FARFUM + LAN-RoP model achieved an overall AUC of 0.83 on the HVDROPDB subset, with strong discrimination for Normal (92.8%) and Plus (100%) but weaker performance for pre-plus (56.0%).

We tested the FARFUM + LAN RoP Model on a Plus-disease subset from the public HVDROPDB dataset. Figure 6; Table 4 show that the model separated Normal and Plus cases well but was weaker on Pre-Plus. The overall AUC on this external set was 0.83. Future work will refine the algorithm to improve Pre-Plus detection, and a broader multi-centre cohort will be pursued in future work to further assess generalisability.

**Table 4:** Shows the results of external validation dataset.

Class	Precision (%)	Recall (%)	F1 score (%)
Normal	96.15	89.29	92.59
Pre-Plus	14.29	8.33	10.53
Plus	26.67	100.00	42.11
Weighted Average	83.55	80.00	80.73
Overall Accuracy		80.00	

## Discussion

We built an AI model for plus disease in ROP using a cross-center dataset and showed that this approach enhances the generalizability of the AI model. The combined dataset outperformed single-center sets, proving that diverse training data helps the model learn universal retinal features instead of population-specific ones. This finding offers a new tool for early ROP detection and has clear clinical value. But Limitations remain, Performance still differed slightly across center, perhaps because retinal structure and lesion patterns vary among groups. The sample size, especially for Han infants, is modest. Although we used data augmentation and transfer learning, overfitting may still occur (32) and robustness needs improvement (33, 34). Future work will enlarge the dataset, with more Han infant images, to boost generalizability. We will test newer architectures and extend tasks to lesion segmentation and quantitative grading of vessel tortuosity and dilation. Multi-center studies will confirm utility in varied clinical settings. We will also explore ways to embed the model in daily workflow to raise efficiency and reduce resource use. In summary, our cross-center AI model improves accuracy and the generalizability. Despite its limits, it lays a solid foundation for future research and clinical use.

## Data Availability

The raw data supporting the conclusions of this article will be made available by the authors, without undue reservation.
