# Supplementary material for: Research on the construction of an AI diagnostic model for plus disease of retinopathy of prematurity based on cross-center fusion datasets
Source: Front Pediatr. 2026 Mar 24;14:1765353. doi: 10.3389/fped.2026.1765353 (PMC13055548; doi:10.3389/fped.2026.1765353)
Supplement: Supplementary file 2 [file Datasheet2.pdf]

Section and item		Checklist item
**Title**		
1	√	Identify the study as developing or evaluating the performance of a multivariable prediction model, the target population, and the outcome to be predicted
**Background**		
2	√	Provide a brief explanation of the healthcare context and rationale for developing or evaluating the performance of all models
**Objectives**		
3	√	Specify the study objectives, including whether the study describes model development, evaluation, or both
**Methods**		
4		Describe the sources of data
5	×	Describe the eligibility criteria and setting where the data were collected Revised
6		Specify the outcome to be predicted by the model, including time horizon of predictions in case of prognostic models
7	√	Specify the type of model, a summary of the model-building steps, and the method for internal validation†
8		Specify the measures used to assess model performance (eg, discrimination, calibration, clinical utility)
**Results**		
9		Report the number of participants and outcome events Revised
10	×	Summarise the predictors in the final model† Revised
11		Report model performance estimates (with confidence intervals) Revised
**Discussion**		
12	√	Give an overall interpretation of the main results
**Registration**		
13	×	Give the registration number and name of the registry or repository Not registered
